# Feminist activist women are masculinized in terms of digit-ratio and social dominance: a possible explanation for the feminist paradox

**DOI:** 10.3389/fpsyg.2014.01011

**Published:** 2014-09-09

**Authors:** Guy Madison, Ulrika Aasa, John Wallert, Michael A. Woodley

**Affiliations:** ^1^Department of Psychology, Umeå University, UmeåSweden; ^2^Department of Community Medicine and Rehabilitation, Umeå University, UmeåSweden; ^3^Center Leo Apostel, Vrije Universiteit Brussel, BrusselsBelgium

**Keywords:** feminism, digit ratio, testosterone, beliefs, dominance, personality, evolutionary psychology, gynephilia

## Abstract

The feminist movement purports to improve conditions for women, and yet only a minority of women in modern societies self-identify as feminists. This is known as the feminist paradox. It has been suggested that feminists exhibit both physiological and psychological characteristics associated with heightened masculinization, which may predispose women for heightened competitiveness, sex-atypical behaviors, and belief in the interchangeability of sex roles. If feminist activists, i.e., those that manufacture the public image of feminism, are indeed masculinized relative to women in general, this might explain why the views and preferences of these two groups are at variance with each other. We measured the 2D:4D digit ratios (collected from both hands) and a personality trait known as dominance (measured with the Directiveness scale) in a sample of women attending a feminist conference. The sample exhibited significantly more masculine 2D:4D and higher dominance ratings than comparison samples representative of women in general, and these variables were furthermore positively correlated for both hands. The feminist paradox might thus to some extent be explained by biological differences between women in general and the activist women who formulate the feminist agenda.

## INTRODUCTION

When people are asked the binary question whether they consider themselves feminist or not, between 5 and 40% identify as such. Among an area-probability sample of 1,460 adults in the USA, 29% of women self-identified as feminists (McCabe, 2005). Among undergraduate students, proportions were 16 (Liss and Erchull, 2010, age range 18–22 years), 19.9 (Myaskovski and Wittig, 1997, age range 19–36 years), 23 (Yoder et al., 2011, age range 18–39 years), and 40% (Abowitz, 2008, age range 18–22 years). Feminist self-identification is positively related to socioeconomic status, however a majority of the daughters of well-educated parents fail to identify as such. Even at a prominent women’s college, 32 out of 70 interview responses were clearly negative to feminism, as coded by the authors (Fox and Auerbach, 1983).

The Merriam-Webster (2013) online dictionary defines feminism as “the theory of the political, economic, and social equality of the sexes” or “organized activity on behalf of women’s rights and interests.” Given that feminism is ostensibly about equality and equal rights, does the low proportion of self-identified feminists indicate that women eschew such issues? This seems not to be the case. Among undergraduate students, 75% of women and 47% of men reported as being very or somewhat concerned about women’s rights (Abowitz, 2008). The way this question is framed leaves open the reason for not being concerned, of course. On the one hand it could be that women’s rights are perceived as satisfactory and equal to those of men, or on the other hand it could be that they are unequal but that this is desirable. More to the point therefore is what people believe actually constitute women’s rights. Rudman and Fairchild (2007) found very high ratings of items reflecting equal rights for the sexes. Mean ratings were 9.15 for women and 8.31 for men on a 10-point scale including items such as “Women and men should have the same sexual freedoms” and “Women should have the same career opportunities that men have.” These results indicate substantial support for equal rights and opportunities.

That three-quarters of women are concerned about women’s rights while less than one-third consider themselves feminists is known as the feminist paradox (Abowitz, 2008, p. 51; Scharff, 2012). Part of the explanation for this paradox might result from the fact that there are many different conceptions of what feminism is or ought to be, and that it lacks a commonly established definition. This is not least of all because the underlying ideologies and opinions of the movement have undergone substantive shifts over the course of the 20th century (Scharff, 2012). Today, several different strands of feminism are currently recognized, the most established ones including Womanism and Liberal, Radical, Socialist, and Cultural Feminism (e.g., Henley et al., 2000). According to instruments supposed to discriminate among them, these five strands are substantially positively correlated (*r* = 0.55–0.78; Henley et al., 1998). Given the relatively poor reliability of these instruments, the true correlations may be different.

On the one hand there is thus a substantive positive manifold of attitudes and beliefs among those who identify as feminists. On the other hand there are many issues that divide groups of self-identified feminists, but which are not captured by the feminist instruments. Zucker (2004) divided 272 women into three types depending on self-identification and endorsement of three so-called cardinal beliefs: (1) “Girls and women have not been treated as well as boys and men in our society,” (2) “Women and men should be paid equally for the same work,” and (3) “Women’s unpaid work should be more socially valued.” She classified 123 women as feminists because they self-identified as such and endorsed all three beliefs, 84 as egalitarians who endorsed all three beliefs but did not identify themselves as feminists, and 65 as non-feminists that did not identify themselves as such and rejected one or more cardinal beliefs. These numbers correspond to 37, 24, and 20% of the total number of respondents, which interestingly contained another 8% who self-identified as feminists but rejected one or more beliefs. Thus, Zucker’s (2004) results demonstrate a rather poor correspondence between self-identifying as a feminist and the beliefs assumed to go together with feminism, since 32% (24 + 8%) of her respondents eschew this very association by supporting one construct but not the other.

This rift between belief in equality and the feminist label raises the question of what exactly it means to self-identify as a feminist. Williams and Wittig (1997) found that major contributing factors to feminist self-labeling were (1) positive evaluation of feminists and (2) previous exposure to feminist thought. However, (3) recognition of discrimination and (4) support of feminist goals (which included items about equality) did not make any unique contributions to the probability of identifying as a feminist. Williams and Wittig comment that men and women are equally likely to support or reject feminist views. “Feminist activism,” however, is associated with women to a greater degree than it is with men and the label “feminist” is attributed to women more often than to men (Williams and Wittig, 1997, p. 893). These authors, as well as Zucker (2004), thus make a distinction between “feminist activism” and “feminist views,” resulting in the somewhat counter-intuitive conclusion that self-labeling as a feminist is related to activism but not necessarily to having feminist views. This suggests the content of the most prolific attitudes and beliefs expressed by “feminist activists” might be quite different from the traditional definitions, such as that from Merriam-Webster. We will thus follow the terminology of Williams and Wittig (1997) and Zucker (2004), recognizing that feminist activists are those who primarily formulate the feminist agenda and contribute to shaping the public image of feminism.

One explanation that has been suggested for why women resent the feminist label is “the overwhelmingly negative portrayal of feminists and feminism by the popular media,” which has depicted “feminists as deviant, man-hating, unrepresentative radicals who were a threat to society” (Zucker, 2004, p. 425). A survey by Scharff (2012) found that amongst a demographically diverse sample of young women sourced from Germany and the UK, 30 out of 40 women rejected feminism as a consequence of their belief that the ideology is unfeminine, associated with lesbianism, and encourages man-hating. Feminism was also found to be strongly associated with unattractiveness and lesbianism by young men and women alike (Rudman and Fairchild, 2007; cf. Fox and Auerbach, 1983). These observations raise the question of whether media misrepresents feminism or not. If it does not, it may be that the feminist movement is in fact no longer limited to the “political, economic, and social equality of the sexes” (Merriam-Webster, 2013). While this may be what mainstream women still consider the core goals of feminism, those active in the movement may have turned to more radical goals. It has for example been reported that there are self-identified feminists who argue for the abolition of the nuclear family, that all men are potential rapists, and so forth (e.g., Stone, 2007). This has been described as a division between Gender feminism and Equity feminism (e.g., Hoff Sommers, 1995), and illustrates that feminism is not a corporation or a state institution that can decide top-down what its policies and goals are. Nor is it an academic discipline, in which the views of scholars with better arguments or data could gain more influence than others. It is therefore difficult to determine what the “correct” representation of feminism is.

The feminist paradox, or the dissociation between feminist self-identification and belief in equality, and the alleged misrepresentation in the media all suggest an underlying inconsistency or conflict, to which we will now turn our attention. While we are wary of misrepresenting contemporary feminism, there seem to be three central and characteristic beliefs: (1) A rejection of the idea of innate psychological differences between the sexes (Pinker, 2002, pp. 340–350; Hyde, 2005; Fine, 2010), which entails the view that sex-roles are arbitrary and interchangeable. (2) Sex differences are social constructions, meaning that they are arbitrary, and a function of social roles, structures, socialization, and attitudes rather than a result of essential and innate differences (e.g., Bussey and Bandura, 1999; Ridgeway, 2001). (3) There are general power imbalances between males and females, that are part of a social and gendered power structure (Williams and Wittig, 1997, p. 895; for a discussion, see Stewart and McDermott, 2004). Based on this model, males are seen as structurally advantaged economically, politically, socially, and sexually (Lyness and Thompson, 1997).

By contrast, evolutionary psychology observes that the basic pattern of psychological differences between the sexes can be explained by their having essentially different innate adaptations associated with, most importantly, women investing considerably more resources into offspring through pregnancy and breast-feeding (e.g., Buss, 2012). Males are more aggressive and risk-taking on average, because these traits have paid off historically in terms of increased fitness, given that male-male aggression and risk-taking in the pursuit of resource acquisition have led to more offspring. This would thus explain why males tend to dominate professions where these traits are necessary for success, such as in the military, business, politics and even crime, where competition is high. Females are on average more sociable and empathic than males, because caring for offspring and negotiating social relations that promote their survival until they reach reproductive age ensured that the mother’s genes live on. Hence women dominate professions where these traits are maximally valued, such as teaching, social work, and in human and veterinary medicine (Lippa, 2010). This social dimension is tapped by one pole of the *people-things* dimension (Prediger, 1982), which exhibits an effect size in excess of 1.0 and ranks amongst the largest inter-sex differences (Lippa, 2010).

Another possible explanation of why feminism represents a minority position amongst women is therefore that the activists who shape feminist attitudes and beliefs are themselves generally more physiologically and psychologically masculinized than is typical for women (Wilson, 2010). This might for example explain their belief in sex-role interchangeability, as they may perceive the behaviors and interests of sex-typical women as incomprehensible and at variance with their own more masculinized preferences in terms of child-rearing and status-seeking. This might then lead them to infer that women in general have been manipulated to become different from themselves by external forces, as embodied by notions of *social constructions* or *gender systems* (e.g., Grossman et al., 1997, p. 84). Zucker (2004) notes that “…many women are exposed to women’s and gender study courses and may find some of the information about sexism compelling, but not all of them go on to engage in women’s right activities to remedy those situations. Perhaps there is something about the willingness to claim the identity that helps people engage in activism” (p. 425). We suggest that this willingness may thus be related to a women’s level of masculinity.

All sex-dimorphic psychological traits vary substantially within each sex and overlap considerably across the sexes. It is therefore meaningful to assess each individual’s level of any sex-dimorphic trait in order to study, for example, relations between such traits or group differences. There is evidence that these differences are in part mediated by hormones, such as androgens, and they can therefore be described as being biologically influenced (Cohen-Bendahan et al., 2005), consistent with evolutionary psychological models (Hines, 2010a,b). For example, testosterone differs between the sexes on the order of 2–4 times in foetal amniotic fluid, two times in pre-adolescents (Dorn et al., 2009), and about 10 times after puberty (Vermeersch et al., 2008). It seems that interests and preferences are more affected by prenatal hormone levels (e.g., Beltz et al., 2011), behavioral tendencies such as aggression more by circulating hormone levels (e.g., Pajer et al., 2006), and abilities such as mental rotation by both prenatal (e.g., Burton et al., 2005) and circulating hormone levels, at least in women (e.g., Hausmann et al., 2000). Reviews of the relationship between hormones and psychological functioning cover several hundred empirical papers that report medium to very large effect sizes (Cohen-Bendahan et al., 2005; Hines, 2010a,b).

We propose the *feminists-as-masculinized-females* theory to account for the host of observations reviewed above, and as a partial explanation for the feminist paradox. Taking the psychology of sex-dimorphic traits and biomarkers into account, this theory makes very specific predictions. Using indicators of prenatal testosterone exposure, feminist activists should exhibit significant evidence of physiological masculinization when compared to a sample of women in general. The most widely used index of prenatal testosterone exposure is 2D:4D, the ratio of the length of the index finger to the ring finger (i.e., Wilson, 1983; Manning and Fink, 2008). Similarly, measures of personality sensitive to masculinity-femininity dimorphism should reveal substantive differences between feminist activists and women in general. This should be especially true of measures that tap components of personality related to aggressiveness, assertiveness, and social dominance.

2D:4D has been studied extensively in the past few decades and comprised more than 450 studies in early 2011 (Voracek, 2011). A large number of psychological variables have been related to 2D:4D, but many results are inconsistent across studies. In general, meta-analyses find many consistent sex differences but few consistent correlations between 2D:4D and psychological variables (Putz et al., 2004; Hönekopp and Watson, 2010). Putz et al. (2004) did include dominance, but unfortunately only for the male participants. Significant correlations have been found for other psychological variables that may be related to dominance. For example, 25% of the variance in endurance running in athletes was explained by 2D:4D (Manning et al., 2007) and teenagers’ physical education grade was negatively associated with 2D:4D (Hönekopp et al., 2006).

Wilson (1983) administered with the help of the UK *Daily Express* newspaper a survey in which he asked female readers to submit self-measurements of 2D:4D along with a self-placement on an item whose ratings ranged from “gentle and feminine” at one pole and “assertive and competitive” at the other. He found that greater assertiveness was associated with a more masculine digit ratio amongst a sample of over 1,000 respondents. A more recent study (Manning and Fink, 2008) also found significant associations between more masculinized 2D:4D in women and a measure of social dominance in a large web-survey of people reporting self-measured 2D:4D. It is not known of course whether the more masculinized women in these studies are in fact on average more feminist in ideological orientation, although Wilson (2010) predicted that they would be. Here, we propose to test this *feminists-as-masculinized-females* theory by comparing women in general with a sample of feminist activists, who belong to the group of women that primarily formulate feminist agenda and contribute to shaping the public image of feminism (Zucker, 2004; Duncan, 2010; Yoder et al., 2011). We hypothesize that feminist activists exhibit a lower (i.e., more masculine) 2D:4D ratio and a higher level of social dominance than women in general, and that these two variables will be negatively correlated.

## MATERIALS AND METHODS

An operational definition of a feminist activist would be someone who engages in organized feminist activity, such as political writing, public debate, and attending feminist conferences and political meetings. Some impression of how common feminist activists might be can be gleaned from the proportion of the population who voted for the Swedish feminist party, Feministiskt Initiativ (Fi), namely 0.7 and 0.4% in the 2006 and 2010 government elections. Subtracting some 20% male voters, one out of somewhere between every 150 and 400 women voted for Fi. To obtain a sample that reflects the normal distribution (*N* = ∼30) therefore requires a selection from 4–12 thousand of the general population. These are of course very rough figures, but it seems safe to say that there be will at least two orders of magnitude between the number of individuals responding to some form of feminism instrument and a valid sample selected on the basis of that instrument. It remains questionable if this approach would be effective, however, because feminism instruments have poor psychometric properties and do not sufficiently represent the more radical beliefs that would distinguish feminist activists from other self-identified feminists (e.g., Henley et al., 1998; Fischer et al., 2000). We therefore recruited our sample directly from the operational definition, that is, attendees at a feminist conference in Sweden. This public one-day event was advertised through posters, flyers, and social internet media, and featured some 20 talks and lectures in several parallel sessions, presented by political and other interest organizations. In the conference hall we set up a table and a sign saying (translated from Swedish) “Answer a few questions and image your hands in exchange for fruit or candy. Your participation is anonymous^1^.” We surmised that any mention of feminism, and the possible connection between feminism and biomarkers in particular, would have deterred attendees from contributing. In order to maximize participation, therefore, we did not ask them if they self-defined as feminists, and did not disclose the purpose of the study.

In total, 35 attendees participated in data collection over the course of the whole conference day, mostly when moving between rooms, in exchange for items of fruit or candy. To ensure anonymity no biographical information was collected, and sex and age were assessed visually (20–45 years). The total number of attendants was estimated at ∼100 over the day and the female-to-male ratio to ∼2/3. Twenty-five of the respondents were female and hence were eligible for inclusion in the analyses, which means that our sample included ∼35% of the female attendees.

Hand scans were obtained with a Canon LiDE 110 high-resolution scanner, set to take grey-scale images with 600 dpi resolution. High reliability finger length measurements were obtained with a software ruler from the scanned images (Allaway et al., 2009). These methods are well established and have the same high reliability as direct measurements with calipers and radiographic measurement, according to meta-analyses (Hönekopp and Watson, 2010). Software ruler measurements were done independently by two research assistants, one of whom was unaware of the research question, the nature of the sample, and the 2D:4D concept and its association with prenatal androgen exposure.

Social dominance was measured with the revised Mark VI version of the Ray Directiveness scale (Ray and Lovejoy, 1984), which includes items like “Are you the sort of person who always likes to get your own way?,” “Do you tend to boss people around?,” “Would you rather take orders than give them?,” and “Would you avoid a job which required you to supervise other people?” The Directiveness scale was employed on the basis that it exhibits a high internal consistency, is balanced against acquiescence, and has been validated in demographically representative samples of the Australian population (Ray and Lovejoy, 1988). Finally, it is a substantial predictor of self-report indicators of masculine versus feminine orientation in both males and females, including the full range of masculine-feminine orientation, probably because it specifically taps “aggressive dominance” (Ray and Lovejoy, 1988). The original instrument was translated to Swedish by way of backtranslation according to established practices (Van de Vijver and Hambleton, 1996). The 14 Directiveness items were presented in a paper questionnaire with a five-step Likert type response scale ranging from “Strongly disagree” to “Strongly agree.”

## RESULTS

One respondent did not answer the questionnaire, and was therefore only included in the 2D:4D analysis. The inter-rater variability of the finger length measurements was 0.042% for 2D and 0.058% for 4D, (∼0.35 mm) and the corresponding inter-rater correlations 0.990 and 0.994, demonstrating very high measurement consistency. Troche et al. (2007) for example, reported a reliability of 0.98 for both hands. To eliminate possible experimenter bias, data from the naive rater were used in the following analyses.

**Table 1** shows the means, SDs, and Ns for all study variables, and demonstrates that the mean 2D:4D is lower than usual for females but close to typical male values (Hampson et al., 2008; Hönekopp and Watson, 2010). We are aware of only three studies that have measured 2D:4D in Swedish women, with 24 (Sanders et al., 2005), 48 (Troche et al., 2007), and 185 participants (von Horn et al., 2010). All of these measured finger lengths directly with calipers, which has been found to yield 2D:4Ds slightly higher for men (0.03) and lower for women (Dressler and Voracek, 2011: 0.03, n.s.) as compared to scans. Such a possible measurement bias is marginal compared to the present difference between the study and comparison groups, however, (≥0.44 for the right hand) and would in any case only have increased the differences between them.

**Table 1:** Descriptive statistics for the study and comparison groups (*N*, Mean, and SD of 2D:4D, and fit of normal distribution).

		*N*	Mean	SD	Kolmogorov–Smirnov d
Women, right hand	Study sample	25	0.9484	0.0176	0.0784 (n.s.)
	Troche et al. (2007)	48	0.992	0.03	
	Sanders et al. (2005)	24	1.01	0.04	
	von Horn et al. (2010)	185	0.98	0.03	
	Aggregate sample	9343	0.9718		

Women, left hand	Study sample	25	0.9510	0.0248	0.1476 (n.s.)
	Troche et al. (2007)	48	0.985	0.030	
	von Horn et al. (2010)	114	0.99	0.03	
	Aggregate sample	8926	0.9717		

Men, right hand	Troche et al. (2007)	48	0.968	0.035	
	Sanders et al. (2005)	24	0.95	0.05	
	von Horn et al. (2010)	114	0.97	0.03	
	Aggregate sample	8000	0.9548	0.0350	

Men, left hand	Troche et al. (2007)	48	0.968	0.035	
	von Horn et al. (2010)	114	0.98	0.03	
	Aggregate sample	7543	0.9569	0.0353	

Directiveness (Ray and Lovejoy, 1984)	Study sample	24	47.0000	8.0757	0.1051 (n.s.)
	Females	126	27.98	7.15	
	Males	88	28.63	6.70	

*All Lilliefors tests for non-normality were non-significant. Values are rounded to 4 decimals or given as in original publications. It was not clear which hand the reported data refer to in Sanders et al. (2005) so they were included only once, for the right hand comparison.*

We primarily compared the means and variances of our study sample with those reported by Troche et al. (2007), because they were smallest on average (Right hand = 0.992; Left hand = 0.985) and therefore constitute the most conservative comparison. This is because combining the Troche et al. (2007) comparison sample with the Sanders et al. (2005) and von Horn et al. (2010) samples would have inflated the differences as their reported 2D:4D were larger (means across hands were 1.01 and 0.98, respectively). In order to reject the possibility that the Swedish comparison samples were unrepresentative, given their small *N*, we also compared the present sample with the grand mean across a large number of studies that used a comparable measurement method. Aggregate estimates of 2D:4D were obtained by computing the means across all studies reviewed by Hönekopp and Watson (2010), 66 for the left hand and 75 for the right hand. Ray and Lovejoy (1984) provide a random population sample comparison for the Directiveness ratings. Tests of normality were conducted for all original data, and exhibited no tendency for non-normality.

**FIGURE 1:**
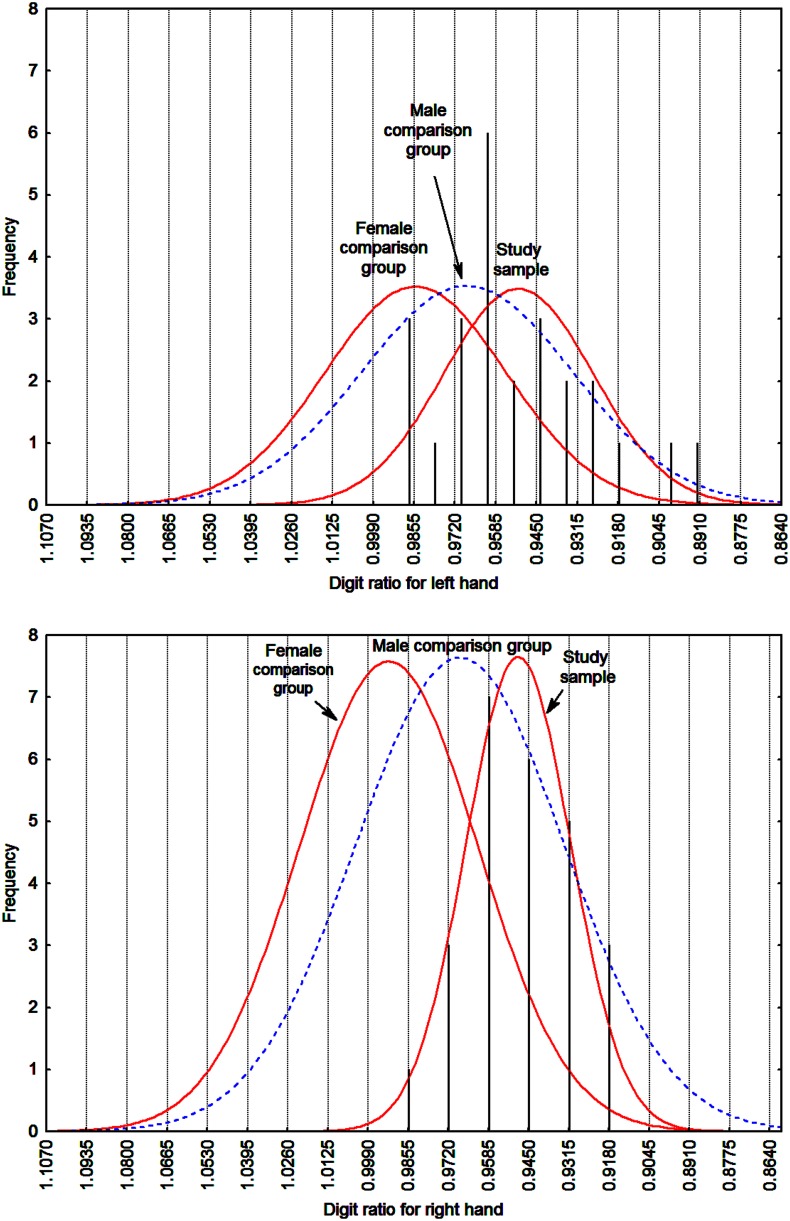
**Histograms of 2D:4D for the left and right hand with fitted continuous Gaussian functions for study sample and both female and male comparison samples (Troche et al., 2007).** Note that the comparison sample functions are scaled to the study sample and are thus unrelated to absolute frequency.

**Table 2** shows pairwise comparisons between the study sample and the national/aggregate samples, tested with Welch’s (1947) *t*-test, which accounts for different variances in the two sample populations, and degrees of freedom calculated with the Welch–Satterthwaite equation. All four (2 hands × 2 comparison samples) differences between the study sample and the typical female samples were in the predicted direction as well as highly statistically significant (α = 0.0005, one-tailed). More importantly, the 2D:4D effect sizes were large, and range from 0.605 to 1.645, with all the lower 0.95 confidence intervals being greater than zero. These results are depicted in **Figure 1**. Comparisons between the study sample and the male comparison samples showed that the feminist activists have a more masculinized 2D:4D ratio than males from the same country. In comparison with the aggregate male sample across nations, however, the difference was significant only for the right hand.

**Table 2:** Analyses of differences between the study sample and the comparison samples: effect size with confidence intervals, percentiles, Pearson’s correlation, Student’s *t*, degrees of freedom, and *p*. “*Across sexes*” denotes the female study sample vs. the male comparison samples.

		Cohen’s *d*	*d* lower CI	*d* upper CI	Percentile	*r*	*t*	df	*p*
Right hand, women	Study sample vs. Troche et al. (2007)	1.645	1.093	2.200	95.00	0.791	7.805	69.88	<0.000001
	Study sample vs. aggregate sample	0.692	0.299	1.084	75.55	0.036	6.593	24.47	<0.000001

Left hand, women	Study sample vs. Troche et al. (2007)	1.200	0.678	1.720	88.50	0.577	5.165	57.52	0.000002
	Study sample vs. aggregate sample	0.605	0.213	0.998	72.75	0.036	4.171	24.26	0.00016

Right hand, across sexes	Study sample vs. Troche et al. (2007)	0.646	0.151	1.141	74.09	0.311	3.176	70.96	0.00102
	Study sample vs. aggregate sample	0.183	-0.209	0.576	57.30	0.010	1.810	24.59	0.0419

Left hand, across sexes	Study sample vs. Troche et al. (2007)	0.532	0.041	1.023	70.27	0.256	2.401	64.34	0.0096
	Study sample vs. aggregate sample	0.166	-0.226	0.558	56.59	0.095	1.179	24.32	0.1247

Directiveness, women	Study sample vs. Ray and Lovejoy (1984)	2.605	2.078	3.132	99.54	0.963	10.763	30.26	<0.000001
Directiveness, across sexes		2.621	2.053	3.187	96.56	0.996	10.225	32.14	<0.000001

**FIGURE 2:**
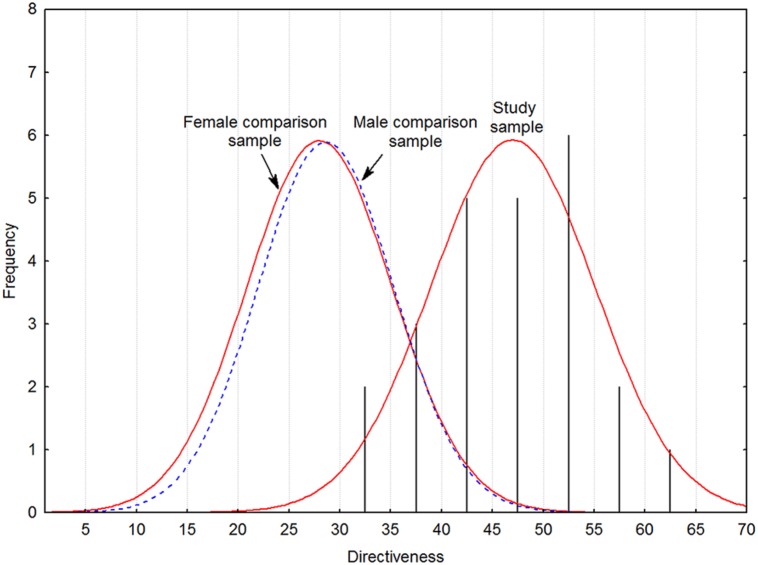
**Histogram of Directiveness scores with fitted continuous Gaussian functions for study sample and both female and male comparison samples (Ray and Lovejoy, 1984).** Note that the comparison sample functions are scaled to the study sample and thus unrelated to absolute frequency.

**Figure 2** details results for Directiveness, demonstrating again that the study sample scores higher than both typical females and typical males. Finally, we examined correlations between left and right hand digit ratios and Directiveness, as summarized in **Table 3**. The reliability of 2D:4D was computed from the inter-rater reliabilities for each finger, i.e., sqrt(0.990 × 0.994) = 0.9920. The Directiveness instrument is unidimensional per the nature of the items, and the standardized Cronbach’s alpha (0.8233, *N* = 24) was therefore taken as an estimate of its reliability (Schmitt, 1996). All correlations corrected for reliability were statistically significant (*p* < 0.05), as were all raw correlations but that between left hand 2D:4D and Directiveness.

**Table 3:** Correlations between digit ratios and directiveness (*N* = 24 for directiveness, and *N* = 25 for 2D:4D).

	Left hand	Right hand	Directiveness
Left hand	(0.9920)	0.5734****	-0.3885*
Right hand	0.5182***	(0.9920)	-0.4964***
Directiveness	-0.3511	-0.4486**	(0.8233)

Alpha coefficients are presented on the diagonal, observed correlations below the diagonal, and correlations corrected for attenuation above the diagonal. *p < 0.05; **p < 0.025; ***p < 0.01; ****p < 0.005 (one-tailed).

In summary, the feminist activist sample had a significantly smaller (i.e., masculinized) 2D:4D ratio than the general female samples. The size of this difference corresponds approximately to a 30% difference in prenatal testosterone/estradiol ratio, which was the index found to have the strongest association with 2D:4D (Lutchmaya et al., 2004). Directiveness self-ratings also exhibit a large and highly significant difference in the predicted direction. It is notable that the feminist activist sample 2D:4D was also more masculinized than those of the male comparison samples, except for the left hand in the aggregate sample (see **Table 2**).

## DISCUSSION

The present study tested whether feminist activists are more physiologically and psychologically masculinized than are women in general. Feminist activists were operationally defined as attendees at a feminist conference, and indices of sex-dimorphism were 2D:4D ratio and Directiveness. Consistent with our specific hypotheses, feminist activists exhibited a significantly more masculinized 2D:4D ratio relative to both Swedish and aggregate comparison groups, a substantially higher level of Directiveness than both the male and female Australian samples, and within-sample correlations between these variables. Consistent with previous research, there were also significant correlations between the hands (Hampson et al., 2008; Manning and Fink, 2008; Butovskaya et al., 2010) and stronger correlations with Directiveness for the right hand (Hönekopp and Watson, 2010). That the results exhibit all the theoretically predicted effects constitutes high *nomological validity* (Cronbach and Meehl, 1955), and the agreement across anthropometric and behavioural measures lends the study a high level of consilience.

Before explicating our conclusions we will consider some possible limitations and sources of error. The present sample was small in absolute numbers, and its reliability and representativeness may be questioned on this ground. However, the effect sizes for the activists – general population differences were between 0.6 and 2.6, depending on the comparison group, and very highly significant. These effect sizes are quite large as psychological dimensions go, and sex differences in personality, for comparison, tend to be 0.1–0.4 (Feingold, 1994). The high levels of statistical significance obtained is of course a product of large mean differences as well as a small variance within each group. The small variance within the feminist activists group attests to its sampling specificity, which leads to the issue of representativity.

As suggested in the method section, the feminist activist population constitutes on the order of 10^-2^ or less of the general population, which for a Swedish city of the size that hosted the present conference corresponds to no more than 500 women. Our sample therefore constitutes ∼5% of the geographically proximate target population, which is in fact a considerably larger proportion than is typically employed for sample-to-target population generalization. We also argue that the voluntary act of visiting the conference, entirely unaffected by any intervention on our part, provides a highly *ecologically valid* selection criterion for the also highly valid operational definition fulfilled by this act.

One possible confounding variable may be the social and contextual conditions of visiting this type of conference, inasmuch as they might have induced attendees to report a higher level of Directiveness than would have been the case under different circumstances. Directiveness forms a behavioral nexus including aggressive, assertive, competitive, and dominance-oriented personality traits, commonly referred to as “bossiness.” This might explain why it is that in comparison to the general female and male populations, the feminist Directiveness mean was in the 99th percentile rather than the 95th, as was the case for right hand 2D:4D. Contextual influence might inflate the Directiveness mean but cannot account for the mean 2D:4D ratio and the within-sample correlation between 2D:4D and Directiveness. It is therefore not possible to wholly attribute the observed Directiveness means to serendipity or to confounding variables.

It could also be argued that the results were somehow confounded by a higher proportion of non-exclusively heterosexual women in the study sample than in the general population. For example, 45% of self-identified feminists in a US sample identified as non-heterosexual, predominantly gynephilious (Liss and Erchull, 2010) as compared to 5.6% in a USA probability sample (Bogaert, 2000), which means that feminists were 4.5 times more likely to be non-exclusively heterosexual. The most recent meta-analysis reported effects sizes from 16 different female samples to have a mean Hedge’s g of 0.230 for the left and 0.285 for the right hand, with a range of -0.242 to 1.873. (Grimbos et al., 2010). Thus, the meta-analytic effect size of female gynephilia is less than half of that related to being a feminist activist, which indicates that even a large proportion of gynephilious women in the present study sample could not explain the level of 2D.4D masculinization reported here. More to the point would be a comparison of the actual 2D:4D values, which are not reported in Grimbos et al. (2010). The nine original studies with the largest effect sizes were therefore scouted for right hand data, which exhibit larger gynephilious–androphilious differences than left hand data. The right hand 2D:4Ds were 0.9786 (Hall and Love, 2003), 0.97 (McFadden and Shubel, 2002), 0.96 (Rahman and Wilson, 2003; Rahman, 2005), 0.969 (Wallien et al., 2008), and 0.963 (Williams et al., 2000) for six of the studies, which corresponds to the effect size range 0.232–0.974 (Grimbos et al., 2010; Figure S1). For the remaining three studies, Putz et al. (2004) did not report 2D:4D data at all, Kraemer et al. (2006) did not report 2D:4D for their non-heterosexual participants, and the dissertation of Tortorice (2002) could not be located. The 2D:4D means for these six groups of gynephilious women are higher, that is, less masculinized, than our study sample. Again, this indicates that the highly masculinized 2D:4D of the feminist activists cannot fully be explained even by a majority of them being gynephilious. Indeed, the study sample 2D:4D was significantly more masculinized (*t* = 3.54, df = 67.98, *p* < 0.0005, *d* = 0.313) than the N-weighted mean of the six gynephilious samples (0.9647, *N* = 327).

Given the wide and cross-disciplinary scope of our theory, we solicited comments from a number of experts in relevant fields. In addition to many insightful suggestions that were easily incorporated, there remain three recurrent themes. One was the representativeness of the study sample, given that we could not measure their agreement with various feminist statements, lest it be even smaller and more self-selected. The other theme is that feminism may mean different things to different people, with the implication that it is not a valid concept or that our use of it lacks validity. Thirdly, concerns were voiced that the present results can be construed as controversial and potentially offensive.

We start with a few disclaimers related to the last point, and note that 2D:4D and Directiveness were analyzed on the group level. Correlations and effect sizes cannot be used in inferring anything about an individual, except in terms of probabilities. Moreover, the target population studied here is not necessarily representative for anyone who sympathizes with feminism or self-identifies as a feminist. As our data pertain to feminist activists, we cannot and do not bring them to bear on women in general. The only connection to women in general consists of figures and statistics based on the works of other scholars cited herein. It would therefore be logically incorrect to infer that, for example, all feminist activists are masculinized or that all groups that are more masculinized are also feminist activists. On the contrary is it highly likely that professions and other activities that benefit from the practitioner being stronger, more aggressive and risk-taking, considered as more masculine traits (e.g., Buss, 2012), would also see a larger proportion of masculinized women among the more successful individuals. Finally, we note that any new knowledge related to any group of individuals may potentially be perceived as offensive by members of that group, but that can obviously not be taken as an argument to suppress such research or to interpret it in a biased fashion.

We concur that definitions of feminism and items in different feminist scales vary (e.g., Williams and Wittig, 1997; Henley et al., 1998; Abowitz, 2008), but inasmuch as this may attenuate the validity of feminism as a concept, that is irrelevant for the present study. This is because the present sample is defined by its behavior, not by its attitudes or specific beliefs, since the crucial connection in our explanation for the feminist paradox is that this group is mainly responsible for the public perception of feminism among the general population, whatever it may be. Conversely, people in general are unlikely to affect the public image of feminism. Nor do they typically consult an encyclopedia before deciding whether to espouse an ideology, let alone to search for alternative ideologies to embrace. Their understanding of the nature of the phenomenon in question is rather based on its public image, conveyed through a range of media. This is in turn based on those individuals who write and speak in public, in the media and in academe, and are generally active in conveying the image of feminism (cf. Hoff Sommers, 1995). The relation between women’s definition of feminism and their self-identification as feminists is therefore irrelevant for the present results, but this interesting issue has been commendably addressed elsewhere (e.g., Fox and Auerbach, 1983; Williams and Wittig, 1997; Rudman and Fairchild, 2007; Abowitz, 2008; Duncan, 2010; Scharff, 2012).

Finally, we consider the argument raised by some colleagues that our conclusions may be invalid because digit ratio and feminist activism are not exclusively associated with one another. The premise seems to be that if both non-feminist female engineers and feminist activists were masculinized, for example, then masculinization is not unique for the feminists, and this relation would therefore be trivial or uninformative. This logic is flawed because the observation that, for example, female engineers might also be masculinized would not invalidate the idea that masculinization might cause either an inclination toward engineering or a feminist interpretation of society – or both. It is therefore a considerable strength of the present design that 2D:4D happens to be a biomarker whose expression during development is determined already before birth (Hönekopp et al., 2007). The notion that environmental variables might affect the biomarker, as is sometimes argued regarding circulating testosterone, is in this case not viable.

In conclusion, the *feminists-as-masculinized-females* theory and these supportive results yield insight into the potential biological origins of feminist beliefs and value systems. These empirical data indicate that Wilson (2010) may have been right to ascribe feministic characteristics to his sample of less feminine females, if the present results generalize to some extent from feminists activists to self-identified feminists and maybe also to women’s support for feminist views. These are important issues for future research based on this theoretical perspective. In any case, our findings shed new light on the feminist paradox and on studies such as that of Scharff (2012) exploring the reasons why women by-and-large eschew feminist ideology.

## AUTHOR CONTRIBUTIONS

Guy Madison and Michael A. Woodley designed the study, John Wallert collected the data, Guy Madison, Ulrika Aasa, and John Wallert analyzed the data, and Guy Madison, Michael A. Woodley, and Ulrika Aasa wrote the paper.

## Conflict of Interest Statement

The authors declare that the research was conducted in the absence of any commercial or financial relationships that could be construed as a potential conflict of interest.

## Footnotes

1 Because participants were anonymous no ethical board approval was required, according to Swedish law (SFS 2003:460, Lag om etikprövning av forskning som avser människor). The hand images were deleted after the finger lengths had been measured to eliminate the possibility that any individual could be identified through unique skin patterns (e.g., fingerprints).
